# Supplementary figures and images for: Encounter rates and engagement times limit the transmission of conjugative plasmids
Source: PLoS Genet. 2025 Feb 7;21(2):e1011560. doi: 10.1371/journal.pgen.1011560 (PMC11828410; doi:10.1371/journal.pgen.1011560)

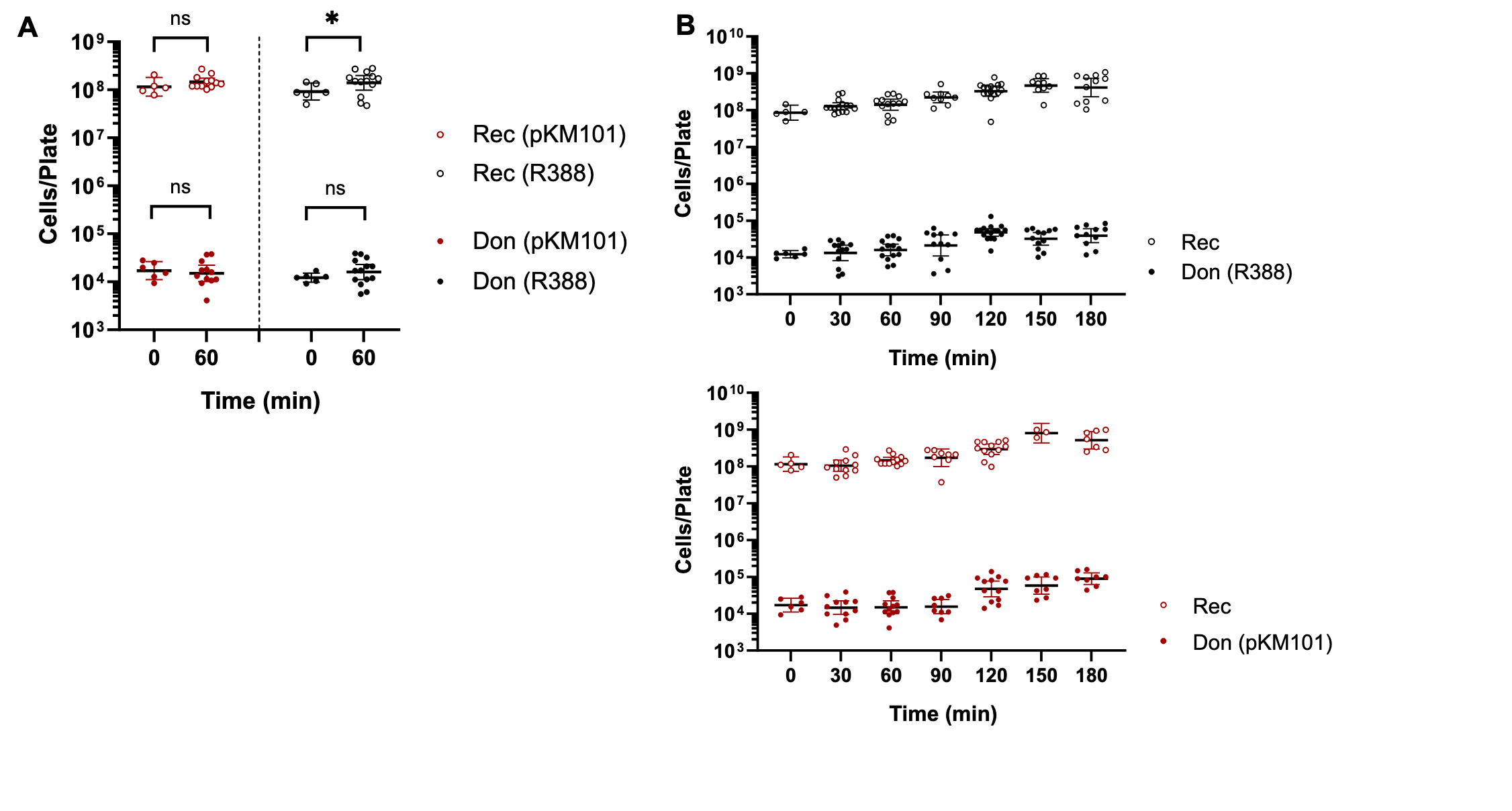

Supplement: S1 Fig — ANOVA testing comparing the four populations at 0 and 60 minutes after mating began gave p > 0.05 in all but R388 Recipients’ growth. B) Growth curves for donor and recipients in matings using plasmid R388 (black circles, upper graph) and pKM101 (red circles, lower graph) fitting showed that doubling times were >60 minutes for all strains analyzed. (TIFF) [file pgen.1011560.s003.tiff]

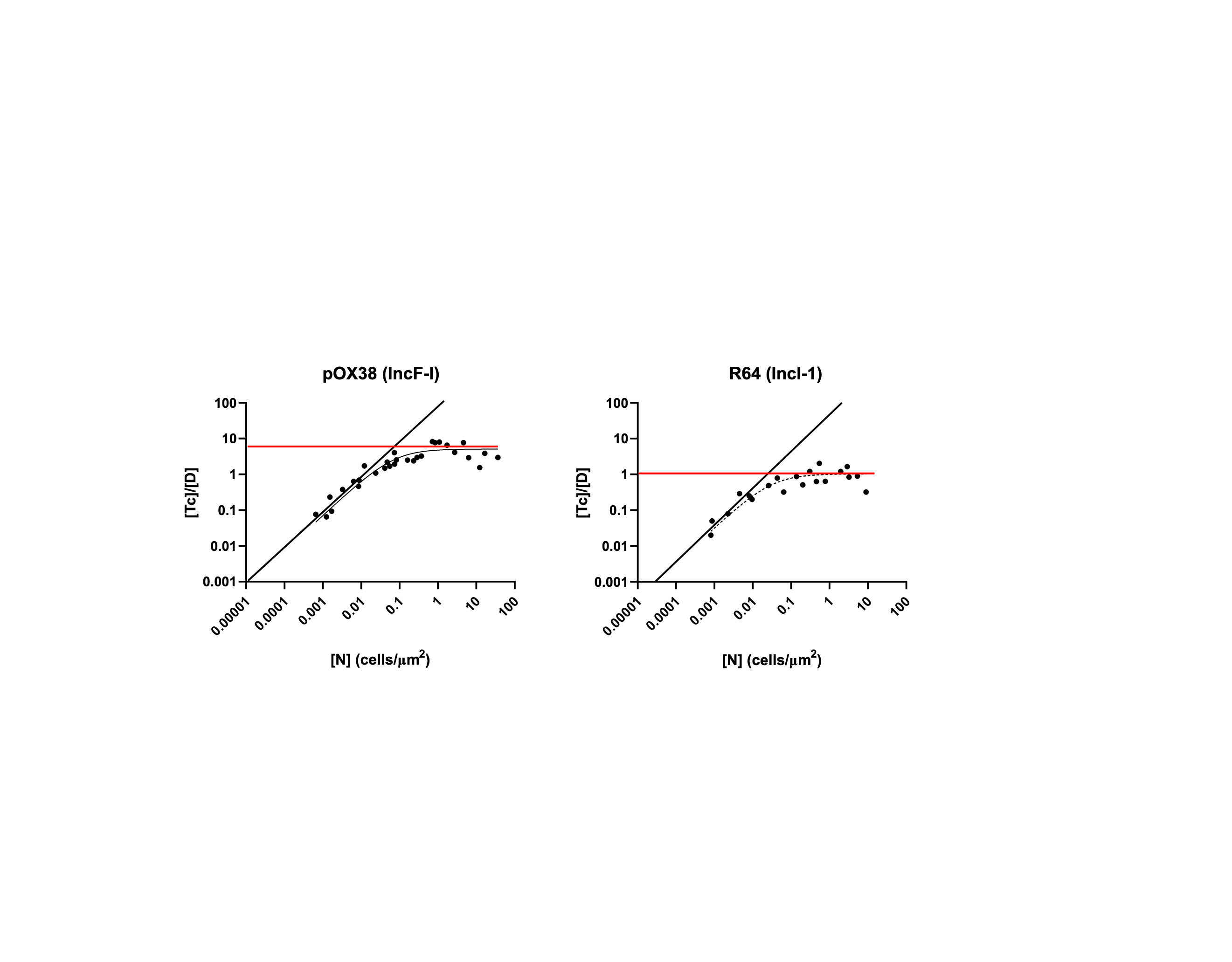

Supplement: S2 Fig — Transconjugants per donor ([T]/[D], y axis) measured at different Recipient densities ([R] ≈ [N], x axis) in E. coli BW27783 mating on solid LB-agar surfaces, 1 Donor to 100 Recipients and 1 h. conjugation time for plasmids pOX38 (IncFI) and R64 (IncI1). As in Fig 1 in the main section, the black line represents the ideal DDT regime, where different kon produce different y-intercepts. The red line represents the FDT regime, where the conjugation efficiency is just 1/τ and the dotted curve corresponds to the fitting to Eq.3. Every black dot corresponds to the average of 3 technical replicates. (TIFF) [file pgen.1011560.s004.tiff]

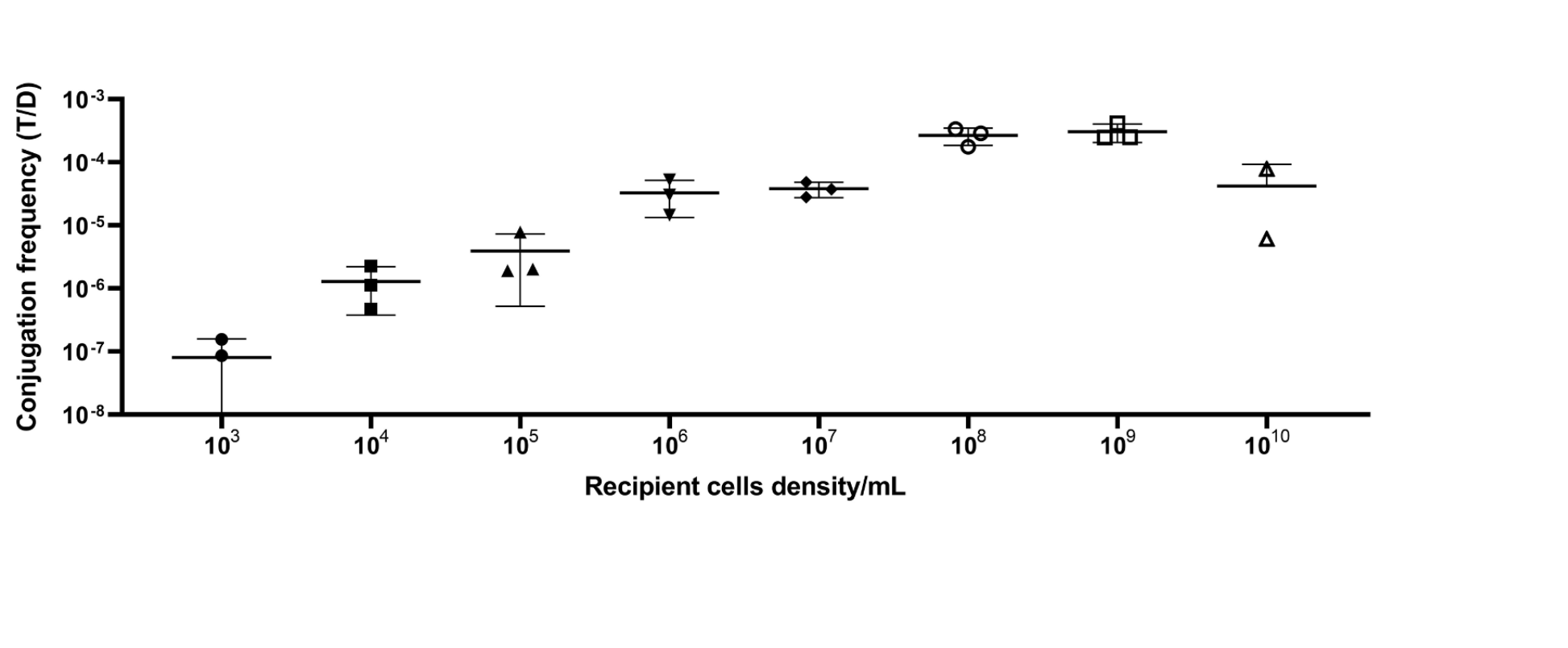

Supplement: S3 Fig — Transconjugants per donor ([T]/[D], y axis) measured at different Recipient densities ([R] ≈ [N], x axis) in E. coli BW27783 mating in liquid LB, 1 Donor to 100 Recipients and 1 h. conjugation. Every dot corresponds to the average of 3 technical replicates. (TIFF) [file pgen.1011560.s005.tiff]

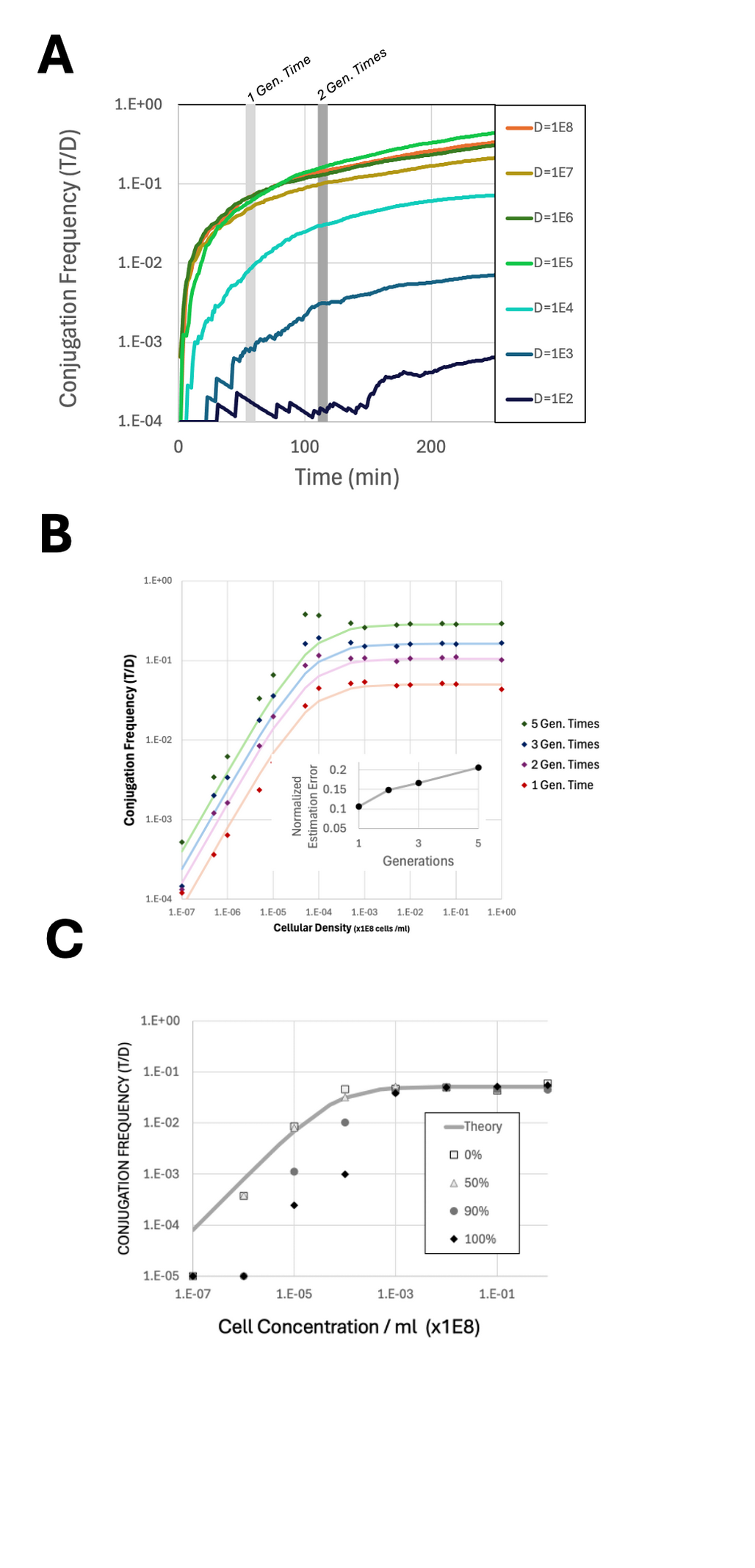

Supplement: S4 Fig — A) Results from stochastic simulations showing the conjugation frequency (T/D, y axis) against time (min, x axis) obtained at different cellular densities (D). Shadowed areas correspond to 1 and 2 generation times. B) Comparison between computational simulations (dots) and theoretical predictions (lines) for the conjugation frequency (x axis, T/D) obtained at different cellular densities (x axis), at different generation times (legend). The normalized estimation error obtained at different generation times is shown in the inner graph. The normalized estimation error was measured as the difference between the conjugation frequency computed and estimated, divided by the average of the two. C) Effect of growth differences between donor and transconjugant cells on the estimation of the conjugation frequency. Computed (dots) and predicted (line) conjugation frequencies (x axis, T/D) obtained at different cell concentrations (x axis), when the transconjugants experience a growth deficit with respect to the donor cells indicated in the legend. As shown in the figure, a significant deviation from the estimated values was observed only for growth deficits >50%. (TIFF) [file pgen.1011560.s006.tiff]

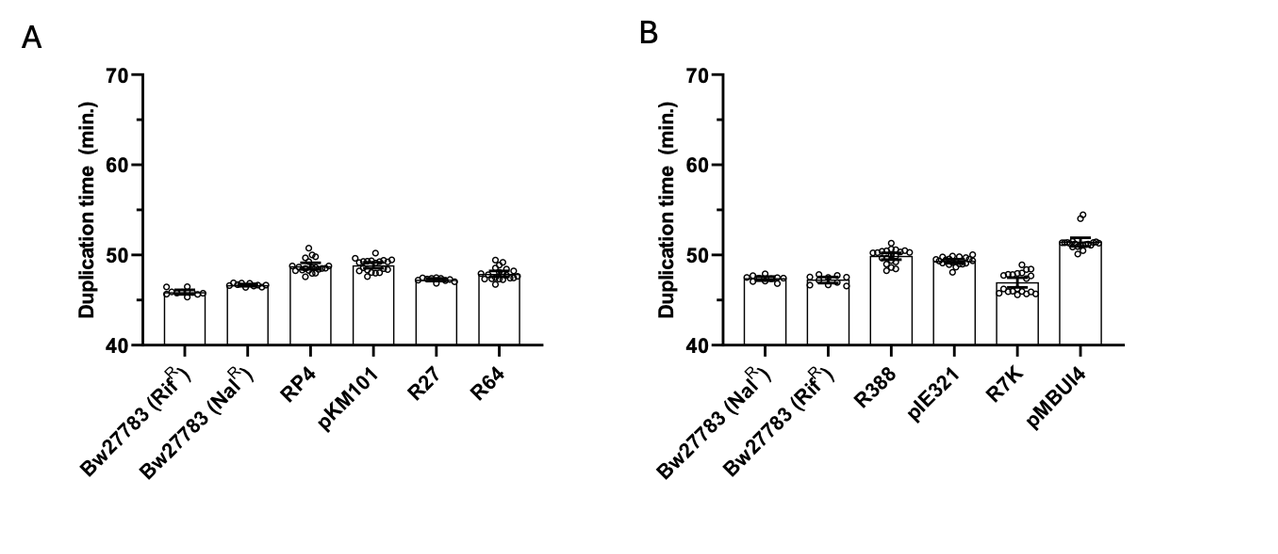

Supplement: S5 Fig — A) Doubling times in LB broth for recipient strains (E. coli Bw27783) and donor cells (Bw27783 containing the plasmids indicated in the legend) for the model plasmids employed in this work. B) Doubling times in LB broth for recipient strains (E. coli Bw27783) and donor cells (Bw27783 containing the plasmids indicated in the legend) for the plasmids from PTU-W employed in this work. (TIFF) [file pgen.1011560.s007.tiff]

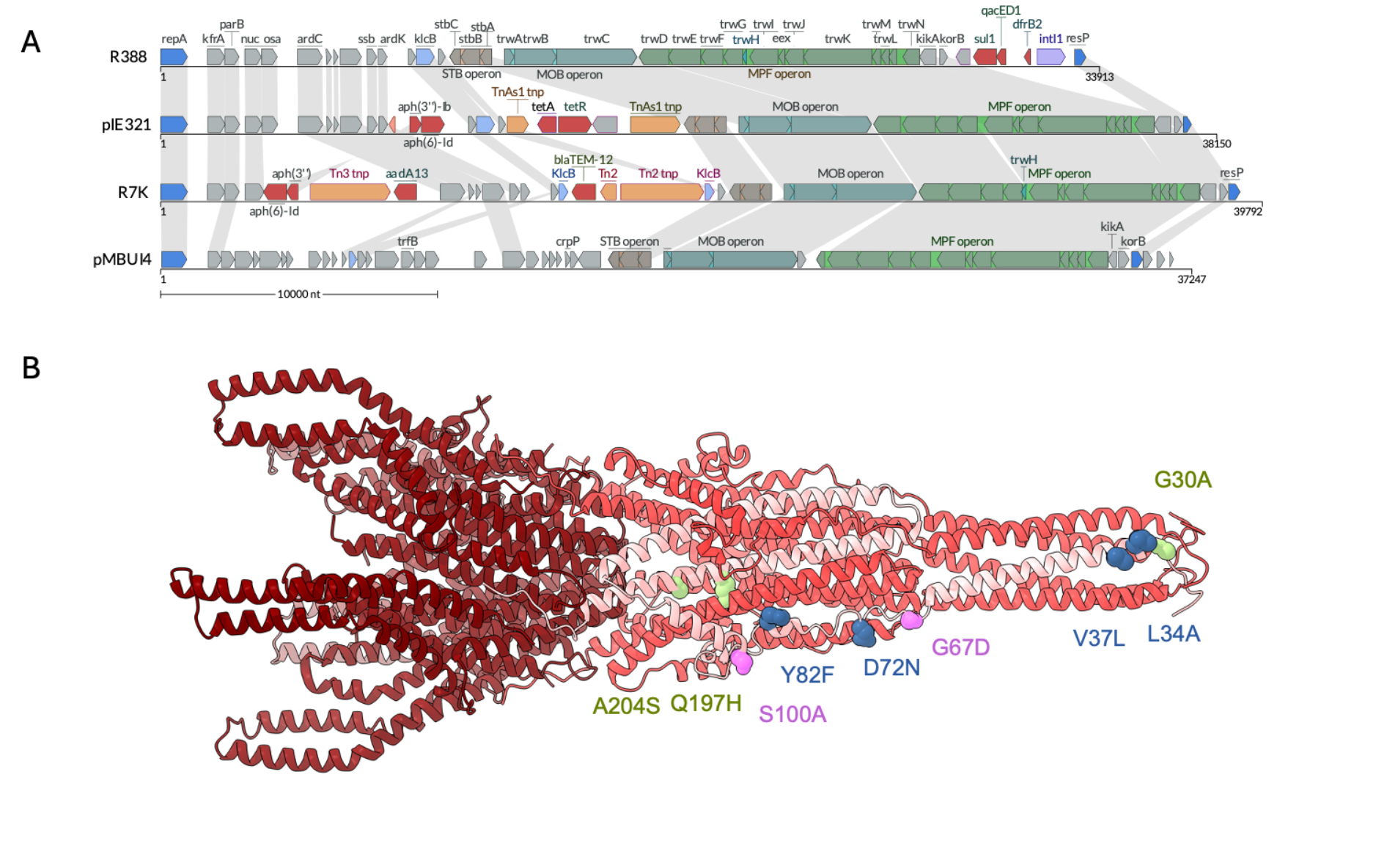

Supplement: S6 Fig — A) Sequence conservation among PTU-W plasmids. Alignment of sequences indicating the synteny on Replication genes (indicated in blue), STB (brown), MOB (light blue) and MPF (green) operons in the 4 plasmids used in Fig 4. Antimicrobial resistance genes are indicated in red, transposases in orange. B) Conservation of the adhesin among PTU-W plasmids. TrwH-TrwG(maroon)-TrwJ(light red) complex from R388 pilus tip, as taken from PDB (https://www.rcsb.org/structure/8RT9). Mutated residues in pIE321, R7K or both are indicated in green, blue and pink, respectively. (TIFF) [file pgen.1011560.s008.tiff]

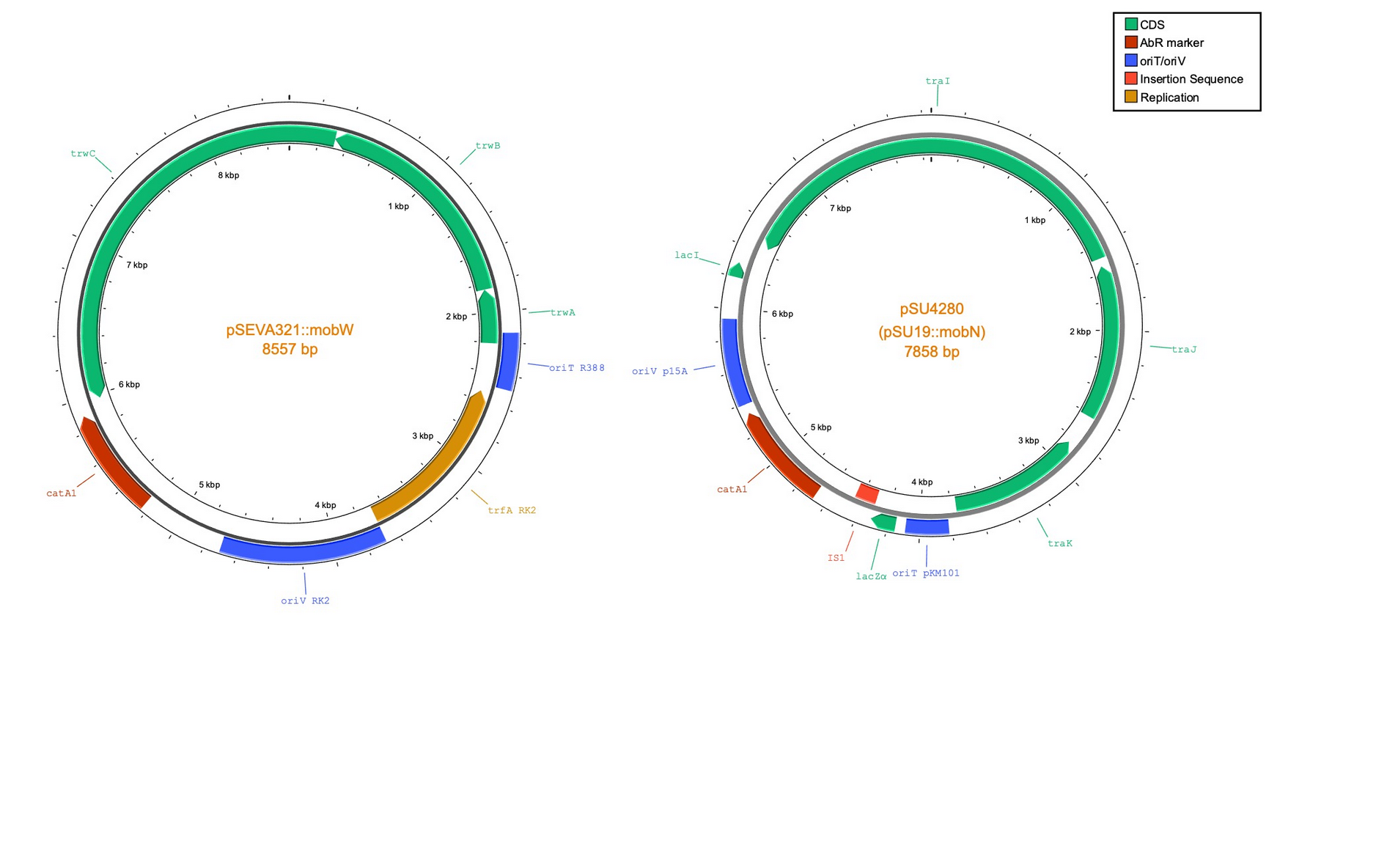

Supplement: S7 Fig — Antimicrobial resistance gene markers are coloured in red, mobilization genes are coloured in green, oriT and oriV sequences are indicated in blue. (TIFF) [file pgen.1011560.s009.tiff]
