# Supplementary material for: Encounter rates and engagement times limit the transmission of conjugative plasmids
Source: PLoS Genet. 2025 Feb 7;21(2):e1011560. doi: 10.1371/journal.pgen.1011560 (PMC11828410; doi:10.1371/journal.pgen.1011560)
Supplement: S1 Calculations — (PDF) [file pgen.1011560.s010.pdf]

SUPPLEMENTARY CALCULATIONS
Encounter rates and engagement times limit the transmission of conjugative plasmids

Jorge Rodriguez-Grande[1], Yelina Ortiz[1], Daniel Garcia-Lopez [1] Maria del Pilar Garcillan[1], Fernando de la Cruz[1] and Raul Fernandez-Lopez[1]

Instituto de Biomedicina y Biotecnologia de Cantabria IBBTEC
Spanish National Research Council CSIC – University of Cantabria
Albert Einstein 22, Santander
Spain.

1.- Deriving the functional response for plasmid mobilization

To obtain the dynamics of mobilization we pose a model with three different types of cells:

D = donors
R= recipients
T= transconjugants

The model assumes that *D* cells encounter *R* in a density-dependent fashion, producing a complex *C*. The conjugative complex may progress to the formation of a transconjugant *T*, or may result in a non-productive mating (by premature termination of the conjugative pair), such that:

$$D + \ R \ \underset{koff}{\overset{kon}{\leftrightarrow}} \ C \ \overset{Kc}{\rightarrow} \ D + T$$

Eq.1

We also will perform our experiments in the regime where *R* >> *D* , thus the number of recipients may be considered a constant. Under these assumptions, the differential equations governing the progression of the population follow:

$$\frac{dD}{dt} = C\ koff + C\ kc - D\ R\ kon$$

Eq. 2

$$\frac{dC}{dt} = D\ R\ kon - C\ koff - C\ kc$$

Eq. 3

$$\frac{dT}{dt} = C\ kc$$

Eq. 4

The system is thus formally equivalent to a Michaelis-Mentem reaction scheme, following the Haldane approximation, we can pose the following:

1) Pseudo-steady state: Given enough time t , formation of *C* complexes will be in steady-state, thus:

$$D\ R\ kon = C\ (koff + kc)$$

Eq. 5

2) Preservation of the total number of *D*: the contribution of vegetative growth during the experiment is considered negligible

$$D(0) = D(t) + C(t)$$

Eq. 6

This allows us to pose an equation which is formally equivalent to that of the MM process under the Haldane approximation:

$$C = \frac{D_0\ R}{\frac{koff + kc}{kon} + R}$$

Eq. 7

$$\frac{dT}{dt} = kc\ C = \frac{D_0\ R\ kc}{\frac{koff + kc}{kon} + R} = \frac{D_0\ R\ kc\ kon}{koff + kc + R\ kon}$$

Eq. 8

Now, we can introduce the average lifetime of C, the time required for the C conjugative complex to resolve into the formation of transconjugants or an abortive reversion to D + R. Such average lifetime (τ) is just 1 / ($koff + kc$). Introducing this factor into Eq. 8 yields

$$\frac{dT}{dt} = kc\ C = \frac{D_0\ R\ kc}{\frac{koff + kc}{kon} + R} = \frac{D_0\ R\ kc}{\frac{1}{\tau\ kon} + R} = \frac{D_0\ R\ kon\ \tau\ kc}{1 + R\ kon\ \tau} = \frac{D_0\ R\ kon}{1 + R\ kon\ \tau}\ \frac{1}{1 + koff/kc}$$

Eq. 9

This Eq can be now directly integrated yielding the following expression for the formation of transconjugants per donor :

$$\frac{T}{D_0} = \left(\frac{R\ kon}{1 + R\ kon\ \tau}\right)\left(\frac{1}{1 + \frac{koff}{kc}}\right)t$$

Eq. 10

Now, in conditions where the formation of a transconjugant is much more likely than the abortion of the conjugative pair, such that kc>> koff Eq 10 simplifies to an expression which is formally equivalent to Holling´s type II functional response:

$$\frac{T}{D_0} = \left(\frac{R\ kon}{1 + R\ kon\ \tau}\right)\ t$$

Eq. 11

In this Eq the transconjugants generated per donor cell depend on two constants: a *kon* parameter that indicates the rate at which donor cells find and attach to recipients, and an effective conjugation time, τ, indicating the time required for a donor-recipient complex to resolve into a new transconjugant. In those cases where the abortion rate of the conjugative plasmid is substantial, the rate of transconjugant formation will be affected by a term which is just the probability of the C complex resulting into the successful formation of a new transconjugant:

$$\frac{T}{D_0} = \left(\frac{R\ kon}{1 + R\ kon\ \tau}\right)\left(\frac{kc}{kc + koff}\right)\ t$$

Eq. 12

2.- Deriving the functional response for plasmid conjugation

The dynamics of plasmid conjugation are significantly more complex than that of mobilization. The newly formed transconjugants can act as donors, a process which is often assumed to involve a lag period. Additionally, transconjugants often exhibit higher conjugation rates than the parental donor population, due to the transitory de-repression of the conjugation machinery after plasmid transmission. An autocatalytic process of this nature, involving lags of variable time and non-linear dynamics, frequently exhibits too complicated dynamics to be faithfully parametrized. We can, however, modify the experimental conditions to simplify the dynamics and obtain the searching rate and the effective conjugation time.

The simplest approximation is to assume that, in conjugative plasmids, the rate of formation of new transconjugants is proportional to the number of donors D_0 and already formed transconjugants (T_t). In these conditions:

$$\frac{\mathrm{dT}}{dt} = \frac{R\,kon}{1 + R\,kon\,\tau}\,(T_t + D_0)$$

Eq. 13

This expression can be directly integrated, yielding:

$$Ln(1 + \frac{T}{D_0}) = \frac{R\,kon}{1 + R\,kon\,\tau}t$$

Eq. 14

For plasmids with low conjugation rates the overall contribution of the newly formed transconjugants is negligible, compared to that of the initial D population. This translates in the convergence of Eq 14 and Eq 11 at low conjugation frequencies. As a rule of thumb, in a plasmid with a conjugation frequency of 0.1 T/D the contribution of secondary conjugation is approximately 0.01 T/D. This means that for most experimental conditions and model plasmids, Eq10 can be directly applied without the introduction of a substantial estimation error. This, however, does not hold for plasmids in which the conjugation frequency observed is higher than 1 T/D (for 1 T/D the error would be 0.3 T/D). This may be the case of highly conjugative plasmids or, more frequently, mating experiments performed for very long times, under a continuous replenishment of nutrients.

3.-Vegetative growth contribution is negligible

In Eq.12 we assumed vegetative growth to yield a negligible contribution to the frequency of conjugation (Fc). Here we will show that this is always the case, as long as the growth rate of T and D is similar.

The FC is defined as the ratio of T to D, such that

$$\frac{dFc}{dt} = \frac{dT_tD_t - dD_tT_t}{dD_t{}^2}$$

Eq. 15

Where

$$\frac{dT}{dt} = \frac{R\ kon}{1 + R\ kon\ \tau}\ (T_t + D_t) + \alpha\ T$$

Eq. 16

And the growth of *D* follows:

$$\frac{dD}{dt} = \alpha\ D$$

Eq. 17

$$D_t = D_0 e^{\alpha t}$$

Eq. 18

In these conditions

$$\frac{dFc}{dt} = \frac{R\ kon}{1 + R\ kon\ \tau}\ \frac{D_t + T_t}{D_t} = \frac{R\ kon}{1 + R\ kon\ \tau}\ (1 + Fc)$$

Eq. 19

Which can be directly integrated

$$Ln(1 + Fc) = \frac{R\ kon}{1 + R\ kon\ \tau} t$$

Eq. 20

4. Testing the approximations using CMEs

The Haldane approximation used to obtain an analytical solution for the model demands that T formation is mostly the byproduct of conjugation , rather than vegetative growth. In fast-growing bacteria such as Enterobacteriaceae, this cannot be guaranteed, thus a quantitative analysis is required. To perform this analysis, we posed the following system of Chemical Master Equations (CMEs):

$$D \xrightarrow{\alpha_D} D + 1 \text{ ; Donor growth}$$
$$T \xrightarrow{\alpha_T} T + 1\text{; Transconjugant growth}$$
$$D, R \xrightarrow{K_{on}} D - 1, R - 1, C_D + 1\text{; Formation of conjugative complex (from D and R)}$$
$$T, R \xrightarrow{K_{on}} T - 1, R - 1, C_T + 1\text{; Formation of conjugative complex (from T and R)}$$

$$C_D \xrightarrow{\boldsymbol{K_c}} T+1\,,D+1\;;\text{ Conjugative complex resolution (D complex)}$$
$$C_T \xrightarrow{\boldsymbol{K_c}} T+2;\;\;;\text{ Conjugative complex resolution (T complex)}$$

In this CME system the growth of R is not considered, since the overabundance of R cells per D (R= 1000 x D aprox.) would result in all computing power directed to R growth events of no consequence to the conjugative dynamics.

Once the CME model was set up, a Matlab script was written to compute the model using Gillespie´s algorithm. The complete code can be found in the repository: https://github.com/IBBTEC/RodriguezGrande24

To test the relative impact of growth on the estimation error of the conjugation frequency, we extracted T/D values at different time points (t= 1 generation, 2 generations, 5 generations etc…) and plotted the computed FCs and the theoretical estimates. As shown in Supplementary Figure 4, the estimation error, measured as $((FC_{Comp} - FC_{The})^{2}/\overline{Fc}^{\,2})^{1/2}$, increased in time, as it was expected. However, at *t*=1 generation, the estimation error was only $\sim$10% of the FC, indicating that the Haldane approximation can be used to estimate conjugation parameters with minimal error.
