# Supplementary material for: Encounter rates and engagement times limit the transmission of conjugative plasmids
Source: PLoS Genet. 2025 Feb 7;21(2):e1011560. doi: 10.1371/journal.pgen.1011560 (PMC11828410; doi:10.1371/journal.pgen.1011560)
Supplement: Attachment — Submitted filename: CoverLetterRodriguezGrande2Submission.pdf [file pgen.1011560.s012.pdf]

Dear editors of PLOS Genetics,

Please find attached a revised version of manuscript PGENETICS-D-24-00879 for your consideration for publication in PLOS Genetics.

We are thankful for the very useful comments and issues raised by both referees; we have tried to address all their concerns. Attached to this document is a point-by-point response to their comments, but the major changes introduced are the following:

- New Data: We have tried to address experimentally, when possible, all issues raised by the ref. 2. We have experimentally determined the growth rate of all model plasmids used in the manuscript. To assess the validity of our approximation, we also created a new computational model based on stochastic chemical master equations that allowed us to compute the relative error introduced by our theoretical framework, and to explore the impact of growth. We have also included the sequence comparisons between the plasmids used, as suggested by both referees. Altogether, this new date is included in four new supplementary figures.

- Editing and presentation issues: We have changed the title as suggested by reviewer #1, and made an effort to convey the key ideas of the paper in a more direct way, as suggested by the referees. In particular, we have substituted the terms "searching rate" and "handling time" , which derived from Ecology, to "encounter rate" and "engagement time", terms that are more often employed in the conjugation literature. Statistical significancy data has been introduced in all figures, as demanded by the refs. and the raw data deposited in two publicly available repositories for review & reuse.

Several of the experiments & figures shown in the revised version have been performed by Daniel Garcia-Lopez, who also participated in the elaboration of the revised manuscript. His direct contribution to the manuscript included the data used for Supplementary Figures 4, 5 and 6. He has been thus added to the authors list, and all other authors approved of this change.

We believe that the issues & comments raised by the reviewers have contributed to a much-improved version of the manuscript, and we hope that we have successfully addressed them all. Thanks a lot for the efforts invested by reviewers and editors. Best regards.

Raul Fernandez- Lopez, on behalf of all authors.

Responses to the reviewers are indicated in red.

Reviewer #1

We are very grateful for all the comments raised by the Reviewer, which primarily address presentation and editing issues.

Reviewer #1 I have to declare that I reviewed a previous version of this manuscript for PLoS Biology. In general, I found the work robust, relevant and interesting. Below, I include my previous comments and suggestions, after removing those that were already corrected by the authors (most of them).

In this manuscript, Rodriguez-Grande and collaborators dissect the conjugations dynamics of plasmids in order to better inform the parametrization of the conjugations process in bacterial population dynamics models. Conjugative plasmids are arguably the main vehicle for the dissemination of antimicrobial resistance (AMR) genes. Given the great threat that AMR presents to public health worldwide, understanding the dissemination of AMR plasmids in bacterial communities is of paramount importance. Most mathematical models assume the principle of mass action to parametrize conjugation, which assumes that the probability of two cells encountering each other and engaging in conjugation is proportional to the densities of both the plasmid-free and plasmid-bearing subpopulations. In this work, the authors show that conjugation is a density-dependent process only at low population density, but it becomes frequency dependent at higher bacterial concentrations. The authors convincingly describe these dynamics and the key parameters that determine them, and they move on to offer the mechanistic insights underlying them. In general, I have really enjoyed reading this manuscript. I think it provides key new results that will be crucial to perform better and more effective models to predict the dissemination of AMR in bacterial populations. Below, I provide a few minor comments and suggestions:

- The title could be somehow more informative and hopefully attract more readers towards the paper, as it reads now is a bit vague.

Agreed. We have changed the title to "*Encounter rates and engagement times limit the transmission of conjugative plasmids*" to specifically indicate the readers the two critical parameters revealed by our analysis as the key determinants of conjugation dynamics. We have also changed the terms searching rate and handling time , which derived from Ecology models, to encounter rates and engagement time, which are likely more representative of the conjugative process.

It would be useful to have a supplementary figure with the schematic

representation of MOBN and MOBW and also to indicate which plasmids are these in Table 3.

Agreed. We have introduced a new supplementary figure (**Supplementary Figure 7**) which complements the description of these plasmids made in Materials and Methods.

Maybe a supplementary figure with an alignment of plasmids R388, pMBUI4, pIE321 and R7K could help. The species of isolation could also be indicated there.

Agreed. We have introduced a new supplementary figure (**Supplementary Figure 6**) which shows the overall conservation between PTU-W plasmids. We have also included a figure showing the mutations present in the adhesin TrwJ, as suggested by Reviewer #2.

Reviewer #2

We thank Rev.#2 for their thorough analysis of the paper. We have tried to address all the issues raised experimentally, when possible, and by implementing a new computational model that allowed us to evaluate the impact of different factors (overall cell density, growth rate) that were not experimentally tractable.

Reviewer #2: Rodriguez-Grande et al have considered how the conjugation dynamics of various plasmids depends on plasmid characteristics (transfer genes) and environmental parameters (cell density, temperature, medium composition). In my opinion, the most interesting and novel aspect is the Holling type II response. However, these findings are only loosely connected to the later parts of the manuscript.

We agree with the reviewer that the latter sections of the manuscript may have appeared somewhat disconnected in the original version, as the exploration of the effects of environmental and transcriptional conditions might seem unrelated to Holling's type II response. Our aim was to identify which factors influenced the encounter rate & the engagement time, the two critical parameters conditioning the dynamics of the response. These are well-known factors that have been previously shown to modulate conjugation frequencies, but this point was probably not clearly stated in the manuscript. We have modified the text to better transmit this idea. (lines 263-266, 280-282 and 284-287 in the main text).

I also have a number of methodological concerns about the assays performed to quantify conjugation, some of which may affect the interpretation of the presented results.

1.) Did the authors perform on-plate mating controls for any of the experiments,

most importantly for Fig 1? Varying the density of cells may well affect the amount of unwanted mating during the selection plating part of the assay (https://link.springer.com/article/10.1007/s00248-024-02427-7 ; https://doi.org/10.1016/j.plasmid.2023.102685). This could lead to biased conjugation rate estimates at higher cell densities. In a related vein, did the authors check the sensitivity and specificity of their selection assay? Is it the same for all considered plasmids?

Although the density of the cells varies in the experiment, the densities at which cells are plated for selection is the same in all cases. Before plating conjugation mixtures are diluted up to x 10^{-8} and different dilutions are plated in selective media. Thus, the cell concentration varies during conjugation, but not during selection. The transconjugants observed at t=0 (Figure 2A) were checked to be due to bona-fide plasmid-transfer events (through antibiogram and plasmid isolation), and not spontaneous mutants to the antibiotic used to select the recipients (which occurred at a frequency of 10^{-8} thus setting our limit of detection).

Regarding the sensitivity and specificity of the antibiotics employed, we tried, when possible, to use the same selecting antibiotics (and recipient /donor strains). In the cases where two selection antibiotics were available (i.e Tc and Ap for pRL443) we checked that both markers yielded similar conjugation frequencies at 1h. In the case of pRL443, we finally opted to use Ap as selection since it was the antibiotic to which pKM101 was resistant, and to prevent the light sensitivity associated to tetracyclines. R388 was selected using trimethoprim, and we previously checked that this antibiotic yields similar conjugation frequencies than Kn-tagged versions (pSU2007).

2.) The introduction to the first results section is misleading. "In a pure FDT, however, the number of T/D cells obtained remains constant regardless of D and R concentrations (Begon et al., 2002)."
I do not think this is generally true. The per capita contact rate increases linearly with density (DDT) or is flat (FDT) - but it is too much to say that the ratio T/D is flat. For instance if the T and D cells have a different growth rate, even in absence of conjugation (whether following DDT or FDT) the ratio T/D would vary over time.
In general, I think the authors should spell out more early and clearly that they use T/D as an estimate of a conjugation rate, and under which conditions this assumption is warranted.

We agree with the referee that the sentence may be misleading, as we meant to say that the number of T/D cells *produced by conjugation* remains constant regardless of D and R concentrations. We have changed the sentence in the main text (line 112).

We felt that the point raised by the reviewer was an important one, since a differential growth between T and D cells may skew the conjugation frequency. Our theoretical model ignores this contribution on purpose, since introducing the

growth rates of D, T and R cells results in a non-linear model that is not analytically tractable. However, an estimation of the error introduced by this approximation was lacking in the previous version of the manuscript.

In the revised version, we built a computational model in which the growth rate of D and T cells can be specifically modified (R cells are in such overabundance that their growth does not contribute to the conjugation dynamics, in our setting.

This stochastic model was computed using the Gillespie algorithm to obtain the number of T and D cells in time (Supplementary Figure 4A), allowing us to determine the conjugation frequency (CF) at different time points. We compared the computed CF (which includes growth) with that inferred by Eq.3 (the analytical approximation that does not take into account growth rates). We plotted them side by side (Supplementary Figure 4B), and measured the estimation error obtained at different time points (1 Gen time, 2 Gen times, 5 Gen times, etc...).

As shown in Supplementary figure 4B, the estimation error of the conjugation frequency is approximately 10% when using a conjugation time of 1 generation (1 h in experimental terms). Transconjugants growing at 50% the rate of their cognate donors do not produce a significant deviation between the theoretical and the computed CF, indicating that Eq3 is a robust approximation of the actual numbers of T and D cells. A 50% growth rate deficit in T cells is a very conservative estimate: previous results showed that T and D cells exhibit an observable growth deficit only during the first generation after conjugation. (Fernandez-Lopez et al, PLOS Genetics, https://doi.org/10.1371/journal.pgen.1004171).

Our computational model also allowed us to to explore which kind of growth deficit should T cells present to skew the estimations of Eq3 significatively. As shown in Supplementary Figure 4C, only T cells that are nearly stagnant (90-100% growth rate deficit) produce a significant deviation in CF. This deviation depends on which cell density is used in the conjugation assay. When saturating cell concentrations are used, even non-growing cells produce a very limited deviation from the estimates of Eq 3.

Altogether, our new data indicated that using short conjugation times (on the order of 1h) eliminates the problem of differential growth rates in the estimation of conjugative rates. This, of course, does not imply that the long-term numbers of T, D and R cells are not influenced by growth. It just demonstrates that the purely conjugative parameters (the encounter rate & engagement time) can be robustly estimated regardless of growth, using short-conjugation times.

We have introduced a paragraph in the main text to convey this idea, a supplementary figure (Supplementary Figure 4) and also a specific section in the supplementary calculations section to show how the estimation error was

measured. The detailed Matlab code to perform these computations can be accessed at https://github.com/IBBTEC/RodriguezGrande24 and it is indicated in the main text.

2') For the short mating assays in Fig 1 T/D may be fine, but for the 3h assays (shown in Figs 2/3/4) Supplementary Fig 1 suggests that the cells are actually growing, possibly at a different rate across different conditions. To exclude that different growth costs of the different constructs are driving these dynamics, it would be advisable to determine the growth rates of the various mutants.

Agreed, but 3h conjugation matings were only used in Figure 2 and Figure 4A. For figure 3, the 3h. indicated in materials and methods correspond to the induction time: the time required for overexpressing the particular genes that are tested in the experiment. The estimation of T and D cells is performed afterwards and conjugation takes place for 1h, as in previous cases.

To evaluate the potential impact of different growth rates in 3h-course experiments (Figure 2 and Figure 4A), we experimentally measured the growth rates of all the plasmids & strains involved. These are included in the new Supplementary Figure 5, which did not show significant differences in growth rate that could explain the faster invasion dynamics of a particular plasmid. Instead, we observed that plasmids that invaded the population faster (pKM101) showed slightly slower growth rates. In the case of PTU-W plasmids, plasmid R7K was the fastest-growing, despite being the slowest in terms of conjugation dynamics. Thus, different growth costs are not correlated with conjugation efficiency at the timescales used in our experiments.

We have introduced a sentence in the manuscript to convey this idea (line 256).

3.) Is it possible to connect the searching rate and handling times to more established metrics for the conjugation rate? It seems the searching rate would be proportional to a bulk conjugation rate on plates?

Unfortunately, it is not possible. Bulk rates measured in conventional assays will be constricted by both the encounter rate and the engagement time, and their relative importance will vary depending on cell density.

In practical terms, we believe that the engagement time is the most significant constraint for conjugations performed under laboratory conditions. In the wild, it would depend on the microbial concentration. In densely packed environments, such as biofilms, the conjugation rate will be also mostly constraint by the engagement time. In scarcely populated environments, it will be the encounter rate the one dominating the process.

Holling´s Type II response is characterized by this double-edged dependency, which means that a single rate is insufficient to parametrize the conjugation efficiency of a given plasmid. It remains to be determined whether both parameters are positively correlated (in which case a single rate may be representative). However, a much higher number of plasmids that those presented here need to be parametrized to demonstrate such correlation.

We have introduced a sentence in the discussion (l.466) to introduce this idea.

4.) Figure 2 shows common conjugation assays, and the relationship to the previous discussion on the Holling Type II response. To assess the effect of the searching and handling time, it would have been nice to compare the dynamics at low and high cell density. What density were these assays performed at?

We totally agree with the referee, and we were eager to perform experiments under controlled densities. The problem we found was that this is only possible in liquid media , using a turbidostat to maintain the cellular density in time. Unfortunately, plasmids conjugating in liquid available to us either belonged to the F-family (which represses transcription of the Tra operon in a cell-density manner) or showed low conjugation frequencies (Supplementary Figure 3). Low conjugation frequencies imply much higher levels of experimental error, thus we opted to perform our invasion assays at high cellular densities, which a) are less confounded by growth effects (Supplementary Figure 4C) and b) offer better CFs and thus lower experimental error.

It is possible, however, to use the computational model to explore the effect of different cellular densities. These can be observed in supplementary figure 4A, which shows the time dynamics of conjugation at different cellular concentrations.

Fig 1: How were the #cells per um determined? The methods section does not seem to suggest that initial time point measurements were taken?
Also, please include error bars from the 3 replicates (either in the main text or a supplementary figure).

We apologize because we did not introduce this information clearly in our first version. The estimation of the #cells per unit surface is taken from previous work (https://doi.org/10.1016/j.plasmid.2012.01.008). We have included a sentence in the main text to specifically state this (l. 128).

Regarding the error bars on Figure 1, since the axis are in logarithmic scale, the error bars are actually smaller than the data points shown in the figure (technical replicates of colony counting fluctuated at much lower levels that inter-experiment variability). The raw data from this figures has been introduced in the data repository to allow a complete assessment of the experimental variability .

Fig. 2:
a) it is confusing that statistical tests were performed for only some but not all comparisons. Were the others not significant? Why did the authors choose not to report tests in panels B and D?

We apologize for the lack of information regarding the comparisons in this figure. In the new version of the figure, we have clearly marked statistically significant differences and the significancy level in all comparisons. As it is now indicated in the legend to the figure, those comparisons that are not marked by asterisks correspond to not statistically significant differences.

b) Which cell density was used for the time course measurements? Where do the observed T/D measurements fall on the Holling Type II response curve? How would the measurements differ if taken at a different point on the curve?

We apologize for leaving out this important piece of information. For the reasons outlined in our response to comment 4) the density employed was on approx. 1 cell /micron, thus at the beginning of the FDT regime. For the experimental reasons also mentioned, we can only estimate using our computational model the effect of performing them at different densities, information that is shown in Supplementary Figure 4A. In general, the time-dynamics of conjugation at different densities were found to be almost parallel, converging to the same threshold as the density increased (reaching the FDT), as theory predicted.

Fig 4: The panels are wrongly referenced in the accompanying text (4A as 4B and vice versa).
We apologize for the mistake. We have corrected this in the revised version.

What is the confidence interval around the fit of searching rate and handling time for these plasmids?

We have introduced now a gray area showing the 95% CI for the theoretical estimates, as well as the CI95 for the encounter rates and engagement times inferred from these fits (Figure 4B).

Other points:
- Please add linenumbers to your future submissions.

We apologize for the missing line numbers, they have been introduced in the new version.

- Fig 1AB: is it intended that both processes lead to the same number of offspring in the same size population? I struggle to understand the message in these figures.

- Suggestion: if the sequences of the PTU-W plasmids are available, it would be interesting to see whether R7K has a TrwJ mutation.

We have extended the figure legend to better explain the process depicted in panes A and B. Regarding the presence of mutations in TrwJ, we introduced a new figure (Supplementary Figure 6B) showing the changes in TrwJ found in R7K and pIE321. As shown in the figure, TrwJ presents several mutations in the different PTU-W plasmids, thus it is possible that changes in the adhesin may play a role in determining the encounter rate & engagement time. However, these plasmids also present extensive mutations in the mobilization and replication regions, compared to R388. Thus it is not possible to associate a particular change with the phenotype observed. We have introduced a sentence in the manuscript to state this (l. 453).

Have all data underlying the figures and results presented in the manuscript been provided?
Large-scale datasets should be made available via a public repository as described in the PLOS Genetics data availability policy, and numerical data that underlies graphs or summary statistics should be provided in spreadsheet form as supporting information.
Reviewer #1: Yes
Reviewer #2: ***No:*** *The data underlying the various figures is not available. The data availability statement reads "available upon request", which did not allow this reviewer to assess them.*

We have deposited the raw data and the matlab code employed in the figures and result shown in the manuscript and they can be found in the following public repositories:

https://osf.io/h3a2d/?view_only=d118a1c874504402bc25d6a94023c029

https://github.com/IBBTEC/RodriguezGrande24
